# Effects of the Pratt pouch model of dispensing nevirapine prophylaxis on HIV exposed infant completion of 6 weeks of prophylaxis in Uganda

**DOI:** 10.1371/journal.pone.0247507

**Published:** 2021-03-10

**Authors:** Edward Bitarakwate, Kim Ashburn, Patrick Kazooba, Ronald Khamasi, Eliab Natumanya, Nicole Herrera, Boaz Owomugisha, Robert A. Malkin, Linda Kisaakye

**Affiliations:** 1 Elizabeth Glaser Pediatric AIDS Foundation (EGPAF), Kampala, Uganda; 2 Elizabeth Glaser Pediatric AIDS Foundation, Washington, DC, United States of America; 3 Biomedical Engineering, Duke University, Durham, North Carolina, United States of America; 4 Uganda Ministry of Health, Kampala, Uganda; University of North Carolina at Chapel Hill, UNITED STATES

## Abstract

**Introduction:**

The innovative Pratt pouch could optimize dispensing nevirapine prophylaxis to HIV-exposed infants in pre-measured single dose pouches to increase completion of the full 6 week infant nevirapine regimen.

**Materials and methods:**

Nineteen health facilities with highest HIV positivity rates among pregnant women across 9 districts in southwest and central Uganda were assigned to control and intervention groups. HIV-positive women enrolled at intervention facilities received pouches filled with premeasured single doses of nevirapine using Uganda national guidelines, which were integrated into the existing drug distribution system. During antenatal care (ANC) women received 14 pouches to cover time until the 6 day postpartum visit, with an additional 8 pouches if women were delayed in returning to the facility, and 28 pouches after delivery. Women enrolled at control facilities received standard nevirapine syrup following delivery for postnatal infant prophylaxis. In a select number of intervention facilities, during ANC, women received all 42 pouches needed to complete the 6 weeks regimen. Medical record data from enrolled women were extracted; interviews with HIV-positive women during postnatal care visits were conducted. Data were collected January to August 2018 (control sites) and October 2019 to February 2020 (intervention sites). Unadjusted and adjusted logistic regression models were used to identify factors associated with facility delivery, postnatal care follow-up visit, and completion of the full 6 weeks infant nevirapine regimen.

**Results:**

Significantly more women in the intervention (n = 320) versus control (n = 340) group had facility delivery (292/316, 92.4% versus 169/340, 49.7%, p<0.0001), postnatal visits within 2 weeks postpartum (295/297, 99.3% versus 133/340, 39.1%, p<0.0001) and reported their infants completing the full 6 weeks infant prophylaxis regimen (299/313, 95.5% versus 210/242, 86.8%, p = 0.0002). Dispensing 42 versus 14 pouches during ANC did not have negative effects on these outcomes. Among out-of-facility deliveries, a higher proportion of infants received nevirapine within 72 hours of birth in the intervention versus control group, 95.8% versus 77.9%. In multivariate models, the intervention group was the only significant factor associated with facility delivery or completion of the full 6 weeks infant prophylaxis.

**Conclusions:**

Use of the Pratt pouch resulted in an increase in HIV-exposed infants completing the full 6weeks prophylaxis regimen and associated benefits including increasing facility delivery and women’s adherence to postnatal care services.

## Introduction

Each year 1.4 million infants in sub-Saharan Africa are born to HIV-positive mothers and exposed to HIV during pregnancy, delivery, and breastfeeding [1]. Globally, Uganda has the fourth highest number of new infections among children. In 2018, 97,722 HIV-exposed infants (HEI) were born in Uganda with an estimated 2,498 becoming HIV-infected [2]. There has been considerable progress among countries in eastern and southern Africa in preventing mother-to-child transmission of HIV, with more than 90% of pregnant women accessing antiretroviral (ARV) medicines in Uganda. This has resulted in a 65% reduction in new HIV infections among children in Uganda since 2010 [3].

This reduction in new HIV infections is hampered by low numbers of HEIs receiving nevirapine prophylaxis. According to the Ugandan Ministry of Health (MoH), only 58% of HEIs receive nevirapine which is distributed at labor and delivery and postnatal care (PNC) services, but not in antenatal care (ANC) [4]. The most current data available in Uganda estimate mother-to-child transmission (MTCT) of HIV to be 1.8% during pregnancy and delivery, and at 2.9% including transmission during breastfeeding [5]. With 37% of infants born outside of health facilities, at least 37,000 HEIs born in 2019 lacked access to infant nevirapine at birth [5]. In addition, since most infants (86%) do not attend the recommended 6 day PNC check-up and do not visit the facility until 6 weeks postpartum for immunizations and an early infant diagnosis test, the majority of these HEIs miss the 6 weeks nevirapine regimen completely [6].

HIV-positive women attending labor and delivery and PNC clinics are provided with a 100 ml bottle of nevirapine and a syringe and health workers must provide the first nevirapine dose to infants and educate mothers how to measure the high-viscosity liquid nevirapine and mark the syringe with the correct dosing line. But, the sticky liquid is difficult to measure and the syringe marking tends to rub off easily [7]. Therefore, most mothers estimate rather than measure correct infant doses. Inconsistent dosing practices across facilities, stock-outs of nevirapine syrup, and busy, overburdened health workers can result in the failure to initiate nevirapine prophylaxis in time for up to 10% of HEIs born in health facilities [4]. In addition, MOH guidelines recommend discarding nevirapine bottles 4 weeks after opening, though mothers are given only 1 bottle for the 6weeks regimen [8]. Identifying methods to increase the number of HEIs who receive nevirapine is a priority for the MOH to reach elimination of pediatric HIV.

The Pratt pouch, an innovative way to deliver nevirapine, is a foilized, polyethylene pouch designed and proven to safely store single nevirapine doses and provide a simple method of dispensing nevirapine suspension to infants [9–13]. Use of the Pratt pouch to provide infant nevirapine doses to HIV-positive pregnant women in ANC, labor and delivery, and PNC offers an innovative way to expand nevirapine prophylaxis coverage in Uganda.

Few previous studies have evaluated the effectiveness of the Pratt pouch as a mechanism to expand infant nevirapine prophylaxis. A study in Tanzania showed that the Pratt pouch can be filled and sealed in real-world settings and can preserve ARV medication for up to 12 months, and mothers are able to open and empty its contents [9]. Duke University trained Tanzanian pharmacists to fill and seal the pouches and mothers were asked to tear and empty its contents into plastic cups approximately the size and shape of an infant’s mouth [10]. Results from a clinical feasibility study of the Pratt pouch in Zambia, showed the use of the pouch lead to a three-fold increase, from 35% to 94%, in the number of HEIs born outside of health facilities accessing ARVs [11]. Data from a feasibility study in Tanzania indicated the pouch was an effective method for dispensing infant nevirapine [12]. A clinical trial in Ecuador demonstrated that the pouch is a highly accurate and an easy-to-use device for delivering multiple liquid oral ARVs to infants and is appropriate for prepackaging ARVs for home use [13].

To improve the number of HEIs accessing infant nevirapine prophylaxis, the Uganda MOH initiated a pilot program in 2017 for developing a model to deliver infant nevirapine using the Pratt pouch in the southwestern region of Uganda. The aim of this study was to evaluate the effect of the Pratt pouch on completion of the infant nevirapine prophylaxis regimen, facility deliveries, and PNC visits within 2 weeks postpartum, in control versus intervention groups.

## Materials and methods

This was a quasi-experimental study using a retrospective review of existing clinical records of HIV-positive pregnant women attending ANC in study facilities and a cross-sectional survey of HIV-positive postpartum women attending PNC services in the same study facilities to evaluate the effects of the Pratt pouch model on infants completing the full 6 weeks infant nevirapine prophylaxis regimen. Study data were collected in control sites from January to August 2018 and in intervention sites from October 2019 to February 2020.

**Fig 1:**
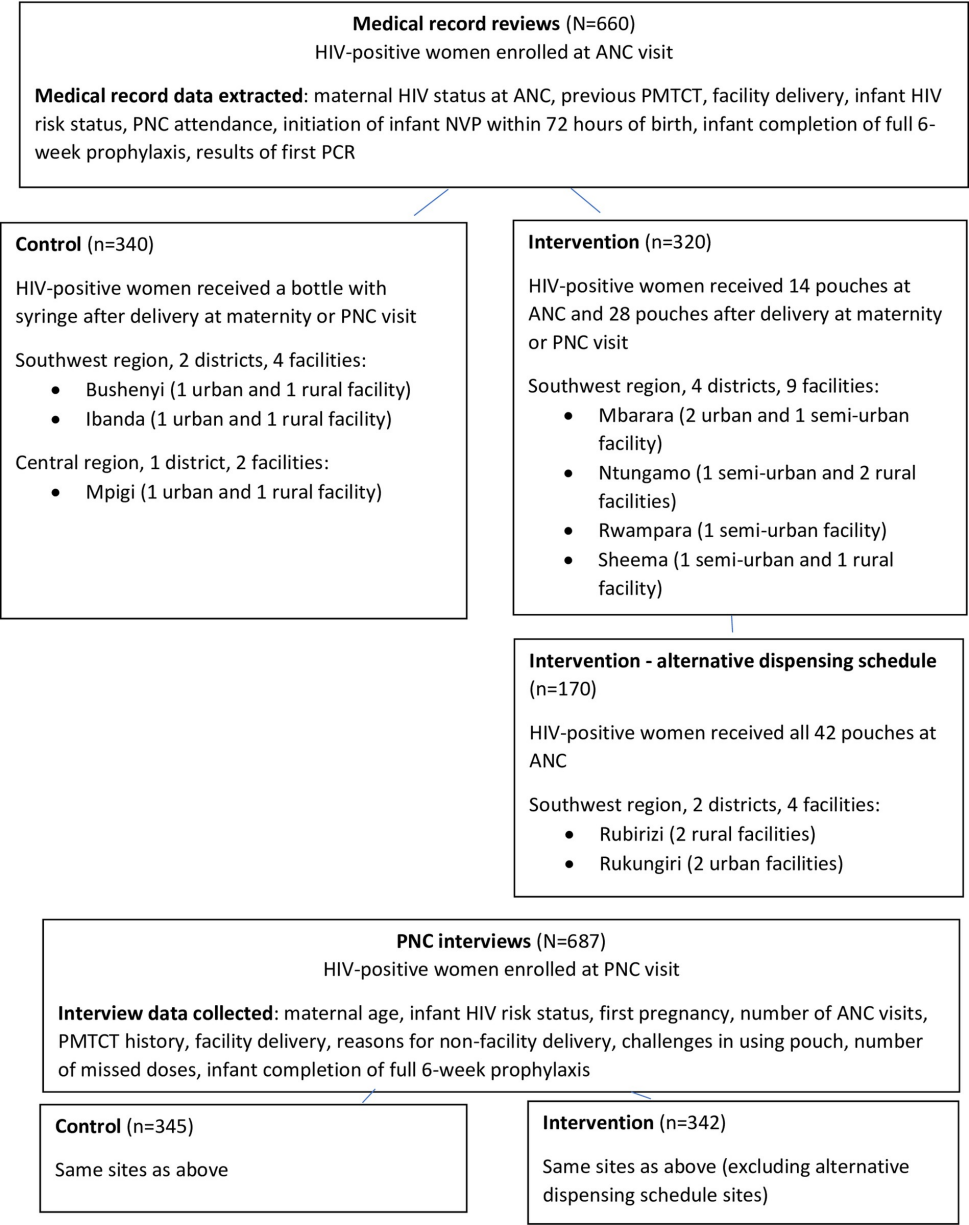
Study enrollment.

The study was conducted in 9 districts selected based on the timing of the roll-out of the Pratt pouch program in health facilities to either implement the Pratt pouch intervention, where infant nevirapine prophylaxis was dispensed using prefilled single dose pouches during ANC (14 pouches) and after delivery (28 pouches), or to continue the standard of care for infant nevirapine dispensing using a bottle with syringe after delivery for infant prophylaxis. In 4 intervention facilities, an alternative distribution schedule was implemented to compare the effects of 14 versus 42 pouches at ANC on outcomes (Fig 1). Districts were selected based on highest HIV prevalence among pregnant women, and included rural, semi-rural, and urban settings. Bushenyi, Ibanda, and Mpigi were selected as control districts and Mbarara, Ntungamo, Rubirizi, Rukingiri, Rwampara, and Sheema as intervention districts. Within each district, 19 health facilities (6 control and 13 intervention) based on highest HIV positivity rate for pregnant women reported in 2016 HIV testing and counseling program data, were selected in the southwest and central regions.

Medical records data were extracted for all HIV-positive women (18 years and older) attending ANC clinics during the study period. Interviews were conducted with HIV-positive women, aged 18 years and older, attending PNC clinic visits at 6 to 18 weeks postpartum during the study period in study facilities. Women whose infants were older than 18 weeks of age were excluded from PNC interviews for the study. Verbal informed consent was obtained from all women participating in PNC interviews for the study and documented in the study enrollment log. PNC interviews were conducted during routine patient care services and the study team felt that verbal informed consent would be less disruptive to service delivery and reduce the potential for breach of confidentiality of study participants. The Institutional Review Boards approved the use of verbal informed consent.

For medical record extraction, a sample of 680 HIV-positive women (340 control group and 340 intervention group) was estimated to allow 80% (0.05) to detect a 15% difference between use of the bottle/syringe versus Pratt pouch in the proportion of infants completing the full 6 weeks infant nevirapine prophylaxis regimen at 6 to 18 weeks postpartum. The medical records of an additional sample of 170 HIV-positive pregnant women were selected from 4 of the 13 facilities in the intervention group (2 rural facilities in Rubirizi and 2 urban facilities in Rukungiri districts) to test whether the distribution of all 42 pouches during ANC is inferior to distribution of 14 pouches at ANC and 28 pouches distributed after delivery.

A similar sample size estimate was used for PNC interviews, 680 HIV-positive women attending PNC at 6 to 18 weeks postpartum at study facilities. Approximately 10% of HEIs were estimated to be high-risk of HIV infection and thus required 12 weeks of nevirapine prophylaxis. Due to the low numbers of these infants, the study was not powered to detect significant differences in the completion of 12 weeks of nevirapine between the Pratt pouch and non-Pratt pouch groups. However, we attempted to interview as many mothers of high-risk infants who attended PNC between 12 and 18 weeks postpartum in all study facilities during the study period as possible.

This protocol was approved by the National AIDS Research Committee in Uganda and registered with the Uganda National Council for Science and Technology, and was approved by the Advarra Institutional in the U.S.

### The Pratt pouch model

Development of the Pratt pouch model involved repackaging nevirapine suspension from the large bottles into Pratt pouches for HEIs. Pouches were filled with single daily doses of liquid nevirapine according to Uganda national guidelines with additional 0.15 mg/mL to accommodate spillage: for infants 2.0 kg to 2.5 kg pouches were filled with 1.15 mg/mL nevirapine; for infants >2.5 kg, 1.65 mg/mL; and for infants 6 to 12 weeks of age, 2.15 mg/mL. At the time of the study, Consolidated Guidelines for the Prevention and Treatment of HIV in Uganda, 2016, required health workers to conduct a risk assessment for MTCT of HIV of the unborn baby at the first ANC visit among all HIV positive pregnant women. High-risk for vertical transmission is indicated when women were newly diagnosed HIV positive in the third trimester or postnatally during the breastfeeding period, received ART for 4 weeks or less before delivery, have unsuppressed viral load (HIV RNA >1,000 copies mg/mL) within 4 weeks of delivery. These high-risk infants receive the standard daily infant NVP doses for the first 6 weeks followed by an increased daily dose to adjust for growth and increased weight of the infant for an additional 6 weeks. High-risk infants returning to the facility postnatally at 6 weeks have their dose adjusted to 2.15 mg/mL to accommodate additional weight gain.

Distribution of Pratt pouches to the participating health facilities was incorporated into the existing national supply chain system. According to MOH guidelines, the standard of care specifies infant nevirapine dispensing after delivery only and not during ANC. The MOH provided guidelines for using the Pratt pouch, which stipulate that HIV-positive pregnant women will be provided with a 2 week (14 pouches) supply of pouches during ANC. The Uganda Sexual and Reproductive Health and Rights Policy Guidelines (2012) provide for postnatal visits at 6 hours, 6 days, and 6 weeks. While women provided with 14 pouches are expected to return to the facility for their 6 day PNC visit, they are given additional nevirapine pouches (8) to cover a return to the clinic within 2 weeks after delivery, when they receive the remaining 28 pouches to complete the 6 weeks prophylaxis. Pouches were delivered in sets of 14 pouches with a patient information leaflet and a plastic bag to store used pouches to return to the facility for proper disposal. At the point of dispensing, ANC women were counselled on dosing. Health worker training materials, and educational and dispensing materials were tailored to the requirements of HIV-positive pregnant and breast-feeding women and their HEIs.

### Data collection and analysis

Individual and clinical data were extracted from ANC, labor and delivery, maternity and PNC records. A trained data manager extracted routine individual level program data from facility ANC, PNC, and early infant diagnosis registers. Information on mode of dispensing nevirapine, the number of pouches, bottles/syringes received during ANC, maternity and PNC, mother’s prevention of mother-to-child transmission (PMTCT) history, infant HIV risk status and initiation of infant nevirapine. Key outcomes were extracted from medical records including facility delivery, PNC within 2 weeks postpartum, completion of 6 and 12 weeks of infant nevirapine regimen, and infant HIV polymerase chain reaction (PCR) results.

Trained nurses and midwives used a structured questionnaire to conduct interviews with consenting eligible mothers. HIV-positive women attending PNC clinic visits at 6 to 18 weeks postpartum were eligible for enrolment into this part of the study. In addition to recording information similar to what was extracted from medical records, the interviews captured information on preference for dispensing method (pouch versus bottle/syringe), challenges in using the pouch, and reasons for out-of-facility deliveries.

All extracted and interview data were anonymized using unique identification numbers. A data manager visited health facilities to verify the extracted data against the facility records. Data was subjected to range and value checks and was verified for consistency and completeness. Any observed errors were resolved. Electronic data was stored in a secure password-protected database only accessible to study staff.

Demographic characteristics at enrollment were summarized using means (standard deviations) or medians (IQR) for continuous variables depending on the distribution, and proportions for categorical variables, stratified by intervention and control groups. Chi square and Fisher’s exact tests of independence for categorical variables and the Wilcoxon-Mann-Whitney test for continuous variables were used to identify any characteristics that differed significantly between study groups. The extracted data were used to identify factors associated with completion of infant nevirapine prophylaxis, facility delivery, and PNC clinic visit within 2 weeks postpartum. Unadjusted bivariate and adjusted multilevel logistic regression models were developed to estimate the association between exposure to Pratt pouch and these key outcomes, in intervention and control groups. To determine multilevel logistic regression models, backwards stepwise regression procedures were used to identify which of the potential variables of interest may affect the key outcome. Once those predictors that were retained in the model were determined, the model was re-run to determine respective odds ratios. PNC interview data were analyzed separately, and results triangulated with the extracted data. A p-value of 0.05% was considered statistically significant. The data analysis for this study was generated using SAS® software, version 9.4 [14].

## Results

A total of 660 medical records were extracted for HIV-positive women aged 18 years and older (320 at intervention and 340 at control sites) and 687 HIV-positive women aged 18 years and older were interviewed at PNC (342 at intervention and 345 at control sites). Delays in distribution of the Pratt pouch lead to slower enrollment in intervention sites and budget constraints resulting in recruitment of 320 women rather than the 340 women estimated in the original sample size estimates. We enrolled 687 women in PNC interviews rather than the original sample size estimate of 680 because we did not want to turn away women at the facility who expressed an interest in participating in the study.

### Participant characteristics

In extracted medical record data, 73.5% of all women had known HIV-positive status at ANC (Table 1). More than half of all women had previously received PMTCT services, with slightly more in the intervention versus control group, although not significantly. Of the 8 infants considered to be high-risk for HIV infection, 7 were in the control group. Facility location differed significantly between control and intervention groups (p<0.0001).

**Table 1:** Demographic characteristics, PMTCT history of HIV-positive women, medical record review.

Variable	Control (N = 340)	Intervention (N = 320)	Total (N = 660)	p-value
	N (%)	N (%)	N (%)	
HIV status in ANC				
Known HIV+	247 (72.6)	238 (74.4)	485 (73.5)	0.62^a^
Newly diagnosed HIV+	93 (27.4)	82 (25.6)	175 (26.5)	
Previous PMTCT				
Yes	172 (50.6)	185 (58.8)	357 (54.1)	0.06^a^
No	168 (49.4)	135 (42.2)	303 (45.9)	
High-risk infant (n = 244 control, n = 306 intervention)				
Yes	7 (2.9)	1 (0.3)	8 (1.5)	0.02^b^
No	237 (97.1)	305 (99.7)	542 (98.5)	
Facility location				
Urban	167 (49.1)	103 (32.2)	270 (40.9)	<0.0001^a^
Semi-urban	0 (0.0)	167 (52.2)	167 (25.3)	
Rural	173 (50.9)	50 (15.6)	223 (33.8)	

^a^ Chi-square test.

^b^Fisher’s exact test.

There were few significant differences between study groups in characteristics of women interviewed at PNC (Table 2). The mean age was 28.8 years. Most women had previous pregnancies and previous PMTCT experience. Of women reporting ANC visits, 41.2% had 4 visits and 40.3% had 5 or more. There was a significantly higher proportion of HEIs at high-risk for vertical transmission of HIV in the control versus intervention group, 2.9% versus 0.6%, p = 0.04. In addition, there was a significant difference in facility location between control and intervention groups, with more rural facilities and no semi-urban facilities in the control group, p<0.0001.

**Table 2:** Demographic characteristics, PMTCT history of HIV positive women, PNC interviews.

Variable	Control	Intervention	Total	p-value
	n = 345	n = 342	N = 687	
	n (%)	n (%)	n (%)	
Woman’s age (years)				
Mean (SD)	28.5 (5.3)	29.2 (5.2)	28.8 (5.2)	0.07^c^
First pregnancy				
Yes	61 (17.7)	61 (17.8)	122 (17.8)	0.96 ^a^
No	284 (82.3)	281 (82.2)	565 (82.2)	
Previous PMTCT (intervention group only)				
Yes	0 (0.0)	250 (73.1)	250 (73.1)	
No	0 (0.0)	92 (26.9)	92 (26.9)	
Number of ANC visits (N = 343 control)				
1	5 (1.5)	3 (0.9)	8 (1.2)	0.75^a^
2	7 (2.0)	5 (1.5)	12 (1.8)	
3	56 (16.3)	51 (14.9)	107 (15.6)	
4	144 (42.0)	138 (40.4)	282 (41.2)	
5 or more	131 (38.2)	145 (42.4)	276 (40.3)	
High-risk infant (N = 344 control)				
Yes	10 (2.9)	2 (0.6)	12 (1.7)	0.04^b^
No	334 (97.1)	340 (99.4)	674 (98.3)	
Facility location				
Urban	174 (50.4)	108 (31.6)	282 (41.0)	<0.0001^a^
Semi-urban	0 (0.0)	155 (45.3)	155 (22.6)	
Rural	171 (49.6)	79 (23.1)	250 (36.4)	

^a^Chi-square test.

^b^Fisher’s exact test.

^c^ T-test.

### Facility delivery, PNC follow up visit, and completion of the 6 weeks infant nevirapine regimen

Table 3 shows data on facility delivery, PNC follow up visit, and infant nevirapine prophylaxis regimen completion extracted from medical records. There were significantly more facility deliveries among women in the intervention versus control group (92.4% versus 49.7%, respectively, p<0.0001). Significantly more infants received nevirapine within 72 hours of birth in the intervention group (97.4% versus 93.1%, p = 0.006); and 17 of the 23 infants who received infant nevirapine more than 72 hours after birth were in the control group. For infants who were born outside of a facility with data on receipt of nevirapine at birth, 60/77 (77.9%) infants in the control group received nevirapine within 72 hours of birth compared to 23/24 (95.8%) infants in the intervention group. Almost all women in the intervention group returned for the PNC visit within 2 weeks postpartum, 99.3%, versus 39.1% of women in the control group (p<0.0001). Of all infants with HIV PCR test results, only 1 in the intervention group had a positive PCR test result. The primary endpoint was full completion of 6 weeks of infant nevirapine prophylaxis. Significantly more infants in the intervention group were reported to complete the full 6 weeks of infant nevirapine prophylaxis than in the control group (95.5% versus 86.8%, p = 0.0002). All of the 8 infants considered high-risk were reported to complete the full 12 weeks of infant nevirapine prophylaxis.

**Table 3:** Facility delivery, PNC visit within 2 weeks postpartum and completion of nevirapine regimen, medical record reviews.

Variable	Control	Intervention	Total	p-value
	N = 340	N = 320	N = 660	
	n (%)	n (%)	n (%)	
Facility delivery (N = 316 intervention)				
Yes	169 (49.7)	292 (92.4)	461 (70.3)	<0.0001 ^a^
No	171 (50.3)	24 (7.6)	195 (29.7)	
Initiation of infant nevirapine at birth N = 246 control; N = 312 intervention)				
Received nevirapine within 72 hours of birth	229 (93.1)	304 (97.4)	533 (95.5)	0.006^a^
Nevirapine received after 72 hours of birth	17 (6.9)	6 (1.9)	23 (4.1)	
No ARVs at birth	0 (0)	2 (0.6)	2 (0.4)	
Initiation of infant nevirapine at birth (out of facility delivery only; N = 77 control; N = 24 intervention)				
Received nevirapine within 72 hours of birth	60 (77.9)	23 (95.8)	83 (82.2)	0.06^b^
Nevirapine received after 72 hours of birth	17 (22.1)	1 (4.2)	18 (17.8)	
No ARVs at birth	0 (0.0)	0 (0.0)	0 (0.0)	
PNC visit within 2 weeks postpartum (N = 297 intervention)				
Yes	133 (39.1)	295 (99.3)	428 (67.2)	<0.0001^b^
No	207 (60.9)	2 (0.7)	209 (32.8)	
Infant age at PNC visit (weeks)				
Median (IQR)	6.0 (6.0–7.0) 6–25	6.0 (6.0–7.0) 4–15	6.0 (6.0–7.0) 4–25	0.02^c^
First PCR test result (N = 215 control, N = 312 intervention)				
Positive	0 (0)	1 (0.3)	1 (0.2)	1.00^b^
Negative	215 (100.0)	311 (99.7)	526 (99.8)	
Completed 6 weeks of infant nevirapine regimen (N = 242 control; N = 313 intervention)				
Yes	210 (86.8)	299 (95.5)	509 (91.7)	0.0002 ^a^
No	32 (13.2)	14 (4.5)	46 (8.3)	
Completed 12 weeks of infant nevirapine regimen (high-risk infants)				
Yes	7 (100)	1 (100)	8 (100)	^d^
No	0 (0)	0 (0)		

^a^ Chi-square test.

^b^Fisher’s exact test.

^c^ Wilcoxon-Mann-Whitney test.

^d^ p-value could not be calculated.

Among women interviewed at PNC, facility deliveries were not significantly different between control and intervention groups (Table 4). None of the women who delivered outside of a facility mentioned the pouch as a reason for non-facility delivery. Nearly all women interviewed received infant nevirapine prophylaxis for her infant, 99.9%. Overall, 96.8% of women preferred the pouch over the bottle and syringe and 95.6% reported no challenges in using the pouch. A small percentage of women mentioned difficulties in opening the pouch (2.1%) or getting the medicine into the baby’s mouth (1.8%).

**Table 4:** Experiences using Pratt pouch among HIV-positive women, PNC interviews.

Variable	Control	Intervention	Total	p-value
	N = 345	N = 342	N = 687	
	n (%)	n (%)	n (%)	
Facility delivery				
Yes	324 (94.2)	320 (93.7)	644 (93.9)	0.74^a^
No	20 (5.8)	22 (6.4)	42 (6.1)	
If non-facility delivery, reason (intervention group only)				
Rapid labor	0 (0.0)	13 (59.1)	13 (59.1)	
No transportation	0 (0.0)	5 (22.7)	5 (22.7)	
No money to pay for transportation	0 (0.0)	2 (9.1)	2 (9.1)	
Preferred traditional birth attendant	0 (0.0)	1 (4.6)	1 (4.6)	
Husband or partner was not available	0 (0.0)	1 (4.6)	1 (4.6)	
Received infant nevirapine				
Yes	344 (99.7)	342 (100.0)	686 (99.9)	
No	1 (0.3)	0 (0.0)	1 (0.15)	
Method of nevirapine dispensing				
Bottle/syringe	344 (100.0)	0 (0.0)	344 (50.1)	
Pratt pouch	0 (0.0)	340 (99.4)	340 (49.6)	
Both	0 (0.0)	2 (0.6)	2 (0.3)	
Number of pouches received at each visit (intervention group only)				
ANC	0 (0.0)	14.2 (2.4)	14.2 (2.4)	
Labor & delivery	0 (0.0)	30.4 (6.3)	30.4 (6.3)	
PNC <2 weeks	0 (0.0)	26.8 (7.1)	26.8 (7.1)	
PNC >2 weeks	0 (0.0)	17.3 (9.5)	17.3 (9.5)	
Preferred method (n = 250 intervention) (intervention group only)				
Bottle/syringe	0 (0)	8 (3.2)		
Pratt pouch	0 (0)	242 (96.8)		
Challenges using pouch (intervention group only, n = 341)				
No challenges	0 (0)	326 (95.6)		
Difficult to open pouch	0 (0)	7 (2.1)		
Could not get medicine into baby’s mouth	0 (0)	6 (1.8)		
Baby did not like taste	0 (0)	1 (0.3)		
Other	0 (0)	1 (0.3)		

^a^ Chi-square test.

### Factors associated with facility delivery, PNC visit, and completion of infant nevirapine regimen

On investigating factors associated with facility delivery and completion of infant nevirapine prophylaxis regimen in the extracted medical record data, the intervention was significantly associated with both outcomes (Tables 5 and 6). Women in the intervention versus control group had significantly greater odds of reporting a facility delivery (odds ratio [OR] 5.45; 95% confidence interval [CI] 3.26, 9.11; p<0.0001) and infant completion of the full 6 weeks of infant nevirapine prophylaxis (OR 3.29; 95% CI 1.69, 6.42; p = 0.0005). Women in urban and rural facilities had significantly lower odds of a facility delivery than women in semi-urban facilities (OR 0.24, 95% CI 0.12, 0.49; p = 0.004) and (OR 0.24; 95% CI 0.11, 0.49; p = 0.005), respectively. In backwards selected adjusted models, the only factor retained in the models was intervention group. Models identifying factors associated with PNC visit within 2 weeks postpartum are not shown because only 2 women in the intervention group did not return for the PNC visit.

**Table 5:** Factors associated with facility delivery among HIV-positive women, medical record reviews.

	Unadjusted			Backwards Selected Model -Adjusted		
Variable	Odds ratio	95% CI	p-value	Odds ratio	95% CI	p-value
Study group						
Intervention	5.45	3.26, 9.11	<0.0001	5.45	3.27, 9.11	<0.0001
Control (ref)						
Infant age at first PNC visit (weeks)	0.96	0.87, 1.06	0.40			
PMTCT history						
Previous PMTCT	1.25	0.81, 1.95	0.32			
No previous PMTCT (ref)						
High-risk infant						
Yes	0.66	0.07, 6.40	0.72			
No (ref)						
HIV diagnosis at ANC						
Newly diagnosed HIV+	0.93	0.57, 1.51	0.75			
Known HIV+ (ref)						
Facility location						
Urban	0.24	0.12, 0.49	0.004			
Rural	0.24	0.11, 049	0.005			
Semi-urban (ref)						

**Table 6:** Factors associated with completion of 6 weeks of infant nevirapine regimen.

	Unadjusted			Backwards Selected Model -Adjusted		
Variable	Odds ratio	95% CI	p-value	Odds ratio	95% CI	p-value
Study group						
Intervention	3.29	1.69, 6.42	0.0005	3.22	1.64, 6.30	0.0006
Control (ref)						
Infant age at first PNC visit (weeks)	1.03	0.78, 1.35	0.86			
PMTCT history						
Previous PMTCT	1.43	0.77, 2.64	0.26			
No previous PMTCT (ref)						
High-risk infant						
Yes	0.27	0.03, 2.68	0.27			
No (ref)						
HIV diagnosis at ANC						
Newly diagnosed HIV+	0.75	0.39, 1.45	0.39			
Known HIV+ (ref)						
Facility location						
Urban	0.70	0.30, 1.61	0.97			
Rural	0.50	0.22, 1.14	0.11			
Semi-urban (ref)						
Facility delivery						
Yes	0.54	0.21, 1.41	0.21			
No (ref)						
PNC visit within 2 weeks postpartum						
Yes	4.54	2.42, 8.50	<0.0001			
No (ref)						

### Alternative distribution schedule

On comparing the alternative dispensing schedule, women receiving all 42 pouches at ANC versus women receiving 14 pouches at ANC and the remaining 28 pouches at the first postpartum visit, no negative effect was found (Table 7). There were significantly more women newly diagnosed with HIV in facilities delivering 42 pouches versus 14 pouches at ANC, 35.6% vs 25.6%, p<0.02. Overall a high proportion of women delivered in a facility, 92.8%, and attended PNC within 2 weeks postpartum, 99.1%, with no significant differences in these outcomes by number of pouches distributed at ANC. A significantly higher proportion of women in the 14-pouch versus the 42-pouch group reported completing the full 6 weeks of infant nevirapine regimen, 95.5% versus 90.2% (p = 0.03).

**Table 7:** Alternate distribution schedule, 14 versus 42 pouches at ANC.

	Intervention 14 pouches	Intervention 42 pouches	Total	p-value
	(N = 320)	(N = 188)	(N = 508)	
	N (%)	N (%)		
Infant age (weeks)				
Median (IQR)	6.0 (6.0–7.0)	6.0 (6.0–7.0)	6.0 (6.0–7.0)	0.01^c^
Previous PMTCT				
Yes	185 (57.8)	94 (50.0)	279 (54.9)	0.09^a^
No	135 (42.2)	94 (50.0)	229 (45.1)	
High-risk infant (N = 306 intervention—14 pouches; N = 156 intervention—42 pouches)				
Yes	1 (0.33)	0.0 (0.0)	1 (0.22)	1.00^b^
No	305 (99.7)	156 (100.0)	461 (99.8)	
HIV status at ANC				
Known HIV+	238 (74.4)	121 (64.4)	359 (70.7)	0.02^a^
Newly diagnosed HIV+	82 (25.6)	67 (35.6)	149 (29.3)	
Facility location (N = 133 intervention– 42 pouches)				
Urban	103 (32.2)	133 (100.0)	236 (52.1)	<0.0001^b^
Semi-urban	167 (52.2)	0 (0.0)	167 (36.9)	
Rural	50 (15.6)	0 (0.0)	50 (11.0)	
Facility delivery (N = 316 intervention– 14 pouches; N = 158 intervention—42 pouches)				
Yes	292 (92.4)	148 (93.7)	440 (92.8)	0.78^b^
No	24 (7.6)	10 (6.3)	34 (7.2)	
PNC visit within 2 weeks postpartum (N = 297 intervention -14 pouches; N = 153 intervention– 42 pouches)				
Yes	295 (99.3)	151 (98.7)	446 (99.1)	0.5^b^
No	2 (0.7)	2 (1.3)	4 (0.89)	
Infant complete 6 weeks of nevirapine (N = 313 intervention -14 pouches; N = 153 –intervention– 42 pouches)				
Yes	299 (95.5)	138 (90.2)	437 (93.8)	0.03^b^
No	14 (4.5)	15 (9.8)	29 (6.2)	
Infant complete 12 weeks of nevirapine (N = 1intervention– 14 pouches; N = 0 intervention-42 pouches)				
Yes	1 (100.0)	0 (0.0)	1 (100.0)	^d^
No	0 (0.0)	0 (0.0)	0 (0.0)	

^a^ Chi-square test.

^b^ Fisher’s exact test.

^c^ Wilcoxon-Mann-Whitney test.

^d^ p-value could not be calculated.

## Discussion

Significantly higher proportions of women who received the Pratt pouch compared to women who used the bottle and syringe had a facility delivery, PNC visit at 6 to 18 weeks postpartum, and reported completion of the full 6 weeks infant nevirapine prophylaxis regimen. Dispensing 42 pouches at ANC, enough to complete the minimum 6 weeks of prophylaxis was not inferior to dispensing a starter set of 14 pouches in ANC followed by an additional 28 pouches at the first PNC visit.

Use of the innovative premeasured, single dose Pratt pouch increased the proportion of infants receiving nevirapine prophylaxis from birth to 6 weeks when the prophylaxis is more effective, compared to delivering infant nevirapine using a bottle and syringe, avoiding many of the complications of measuring the proper dosage and wasting medication. Delivering infant nevirapine using the pouch encouraged women to initiate infant nevirapine after delivery, improving access to nevirapine for out-of-facility births, to deliver in a health facility and to attend PNC visit within 2 weeks of delivery. Our study results suggest that the Pratt pouch is not only a clinically appropriate medicine delivery system but is very effective in reducing the risk of MTCT at birth and postnatally, particularly given the many challenges with dispensing infant nevirapine prophylaxis in the Ugandan context and in other similar settings.

Receiving infant nevirapine at ANC did not have a negative impact on women accessing maternal and child health care services. Less time may have been required during the ANC visit to educate women on how to use the Pratt pouch versus the more complicated instructions for using the bottle and syringe, which could have allowed health providers to focus on counseling mothers about the benefits of both facility delivery and the importance of returning to the facility for PNC.

Dispensing infant nevirapine during ANC had a positive effect on the numbers of infants receiving nevirapine within the first 72 hours after birth. Women in the control group had a higher proportion of out-of-facility deliveries and fewer women in this group reported infants receiving nevirapine prophylaxis immediately after birth. The standard of care in Uganda is to provide infant nevirapine postnatally either at delivery or during PNC. Taking advantage of the high proportion of women in Uganda who have a first ANC visit to dispense infant nevirapine would help ensure infants receive nevirapine immediately after birth, irrespective of where the infants are delivered. Further, delivering all 42 pouches during ANC did not negatively affect facility delivery or attending PNC follow up visits. Significantly, among infants delivered out-of-facility, more infants received nevirapine in the first 72 hours if they received the Pratt pouch versus infants that received the bottle and syringe.

The Pratt pouch uses single premeasured doses, limiting the possibility of overdosing infants or wasting medication. We did not measure spillage of medicine, but less than 2% (6/340) of women interviewed at PNC reported challenges in getting the medicine into the baby’s mouth. Findings in a study from Zambia showed little reported spilling of medication or other challenges in using the pouch to dispense nevirapine prophylaxis (10). A qualitative assessment of women’s experiences with the Pratt pouch, identifying any difficulties encountered in using or storing the pouches would be helpful to guide health providers in supporting and counseling women receiving the Pratt pouch.

Feasibility data were not collected as part of this study. While results show positive effects of the Pratt pouch on important outcomes along the PMTCT cascade, understanding the cost of optimizing the existing health system to repackage nevirapine into single dose pouches is an important missing piece. In Uganda, costs were incurred in the renovation of a site where repackaging could be done according to existing national regulations and licensing requirements, as well as the equipment, staffing, and training involved in this process. Alternatives to establishing repackaging facilities at the facility- or country-level may include identification of an existing facility in the region where systems for repackaging of drugs already exist or convincing a manufacturer to repackage the medication.

Relevant to these results is the increasing global problem of HIV drug resistance, particularly for non-nucleoside reverse-transcriptase inhibitors (NNRTI), the drug class that includes nevirapine. According to the WHO, surveillance survey data from Uganda report NNRTI pretreatment drug resistance in nearly 40% of newly HIV diagnosed infants aged 18 months and younger who were exposed to maternal or neonatal NNRTI ARV prophylaxis [15]. In response to the high rates of ARV drug resistance, Uganda has revised national guidelines to increase access to non-NNRTI treatment formulations, specifically making dolutegravir the preferred first-line ARV drug for children weighing 20 kgs or more and for adults, including women and adolescents of childbearing age. The current high rates of pretreatment ARV drug resistance in Uganda highlights the need for access to new non-NNRTI formulations and treatment optimization for pediatrics; and reinforces the critical importance of ARV drug resistance testing for ARV-exposed infants to ensure optimal ARV drugs are selected for first-line treatment.

There are several limitations to this study. Completion of the full 6 weeks of infant nevirapine was self-reported by women during the PNC visit, as documented in the clinical records. PNC interview data was collected among women who already practice good health-seeking behavior, given high proportions of women in control, 94.2%, and intervention, 93.7%, groups reporting a facility delivery. The control group included only facilities in rural and urban settings, and none in semi-urban settings. Most of the small number of high-risk infants were in the control group. This sampling may have introduced some bias because of the challenges in accessing maternity and HIV services, including HIV testing and ART services, in rural areas. However, facility location was not significantly associated with facility delivery or infants completing the full 6 weeks prophylaxis regimen in the multivariate models. There was a lag of several months between the time data was extracted at control and intervention sites, due to delays in distributing pouches to participating health facilities. It is unlikely that this delay influenced the results significantly, and potentially reduced the possibility of contamination where women at control sites may have switched to intervention sites to access the pouch or receive services. Only 2 women in our study received both the bottle/syringe and the Pratt pouch during ANC, maternity, or PNC clinic visits.

## Conclusions

The Pratt pouch significantly improved reported completion of the full 6 weeks of infant nevirapine prophylaxis among HEIs, facility deliveries, and women returning for PNC visits within 2 weeks of delivery. Optimizing the dispensing of infant nevirapine at ANC could ensure more HEIs receive nevirapine prophylaxis within 72 hours of birth, regardless of where delivery occurs. Cost data of repackaging nevirapine suspension into single dose nevirapine Pratt pouches would be helpful for understanding the feasibility of this model in practice.

## Supporting information

S1 Appendix Pratt pouch effectiveness study PNC interview form (English). (PDF)

S2 Appendix Pratt pouch effectiveness study PNC interview form (Rukiga/Ruyankole). (PDF)

S1 Dataset (ZIP)
