# Supplementary material for: Effects of the Pratt pouch model of dispensing nevirapine prophylaxis on HIV exposed infant completion of 6 weeks of prophylaxis in Uganda
Source: PLoS One. 2021 Mar 10;16(3):e0247507. doi: 10.1371/journal.pone.0247507 (PMC7946283; doi:10.1371/journal.pone.0247507)
Supplement: S1 Appendix — (PDF) [file pone.0247507.s001.pdf]

Participant ID ______________________________

Evaluating the Effectiveness of the Pratt Pouch Model of Dispensing Nevirapine Suspension on HIV Exposed Infants Completion of 6 Weeks of Prophylaxis in Uganda

PNC Interview/Data Collection Form

<table>
<tr><th></th><th>Question</th><th>Response</th><th>Skips</th></tr>
<tr><td></td><td colspan="2">Section 1: Background</td><td></td></tr>
<tr><td>1</td><td>Interviewer code</td><td></td><td></td></tr>
<tr><td>2</td><td>Date of interview</td><td>_ _/_ _/_ _ _ _
(dd/mm/yyyy)</td><td></td></tr>
<tr><td>3</td><td>Mother’s age</td><td>_________years</td><td></td></tr>
<tr><td>4</td><td>Infant’s age</td><td>_______months</td><td></td></tr>
<tr><td>5</td><td>Number of ANC visits</td><td>____ 1 visit
____ 2 visits
____ 3 visits
____ 4 visits
____ > 4 visits
_______None</td><td></td></tr>
<tr><td>6</td><td>Year of mother’s HIV diagnosis</td><td>_ _ _ _</td><td></td></tr>
<tr><td>7</td><td>First pregnancy</td><td>_____ Yes
_____No</td><td></td></tr>
<tr><td></td><td colspan="2">Section 2: Nevirapine Distribution</td><td></td></tr>
<tr><td>8</td><td>Did the mother receive Nevirapine for PMTCT?</td><td>_____ Yes
_____No</td><td>If no, stop interview</td></tr>
<tr><td>9</td><td>Did she receive Nevirapine in a bottle with a syringe or Pratt pouch or both?</td><td>____ Bottle and syringe
____ Pratt pouch
____ Both
____ Tablets</td><td>If Tablets, Stop interview</td></tr>
<tr><td>10</td><td>How many bottles/pouches did she receive at each of the time points?</td><td colspan="2"><table><tr><th></th><th>ANC</th><th>L&D</th><th>PNC ≤2 weeks</th><th>PNC > 2 weeks</th></tr><tr><td>Bottle</td><td></td><td></td><td></td><td></td></tr><tr><td>Pouches</td><td></td><td></td><td></td><td></td></tr></table></td></tr>
<tr><td>11</td><td>Did the infant complete 6 weeks of NVP?</td><td>_____ Yes
_____No</td><td></td></tr>
</table>

12	Does the infant meet the criteria for high risk of transmission?	____ Yes<br>____No	**If no, skp to Q14**
13	Did the infant complete 12 weeks of NVP?	____ Yes<br>____No	
	**ASK THE MOTHER THE FOLLOWING QUESTIONS**		
14	Did the nurse talk with you or give you information about how to give the HIV medicine to your baby?	____ Yes<br>____No	
15	Did you deliver your baby in a health facility?	____ Yes<br>____No	**If yes, skip to q17**
16	If you did not delivery your baby in a health facility, what is the main reason why not?<br><br>*Mark only one answer.*	____ Rapid labor, no time to reach the facility<br>____ No transportation<br>____ No money to pay for fees/ transportation<br>____ Preferred traditional birth attendant<br>____ Husband or partner was not available<br>____ Received the Pratt pouch<br>____ Husband/ partner did not allow<br>___ Other<br>Specify_________________________	
17	Did you give your baby any of the medicine that was given to you in the (bottle/ pouches/both)?	____ Yes<br>____No	If no, **skip to q19**
18	If so, for how many days did you give the baby the medicine?	____ (days)	
19	How many days did you miss giving the baby the medicine?	____ (days)	
	**Check response in q9. If bottle only, end interview**		
20	How many unopened pouches do you have left?	______ (number of pouches)<br>____ Unknown	
21	Have you been through PMTCT before?	____ Yes<br>____No	
22	What method of receiving the medicine for the baby do you like better?	_____ Bottle and syringe<br>_____ Pouch	
23	Were there any difficulties you had with using the pouch?	___ None<br>___ Difficult to open pouch<br>___ Instructions too difficult<br>___ Forgot instructions	

		___ Storage was a problem<br>___ Bulk of pouches<br>___ Could not get medicine into baby's mouth<br>___ Giving medicine interrupted breastfeeding<br>___ Baby did not like taste<br>___ Other<br>Specify__________________________	

Thank you for participating in this interview!

Interviewer Signature: ____ Date:
