# Supplementary material for: Effects of the Pratt pouch model of dispensing nevirapine prophylaxis on HIV exposed infant completion of 6 weeks of prophylaxis in Uganda
Source: PLoS One. 2021 Mar 10;16(3):e0247507. doi: 10.1371/journal.pone.0247507 (PMC7946283; doi:10.1371/journal.pone.0247507)
Supplement: S2 Appendix — (PDF) [file pone.0247507.s002.pdf]

Ennamba y’omuntu oyetabire omukucondooza…………..

Okucondooza ahankora ya Pratt Pouch omuri Uganda. Okuhereza abaana abaine wiiki 6 abari omubuzibu bw’okukwaatwa akakooka ka sirimu omubazi gwa Nevirapine, enkora egi n’ekora ninga tekukora

Ebibuuzo bya PNC/Foomu (form) erikworeka ebyagarukwamu

	Ekibuuzo	Ekyagarukwamu	Eky’okukora
	**Ekicweeka ky’okubanza: Entandikiro**		
1	Ennamba y’endagiriro y’omuntu orikubuzibwa ebibuuzo		
2	Ebiro by’okweezi by’okubuziibwa ebibuuzo	_ _/_ _/_ _ _ _<br>(dd/mm/yyyy)	
3	Emyaaka y’omwaana	_________ (emyaaka)	
4	Emyaaka y’omwaana	_________(omwezi)	
5	Emirundi y’okutayayira ANC	___omurundi gumwe<br>___emirundi ebiri<br>___emirundi eshatu<br>___emirundi ena<br>___emirundi 4 n’aheiguru<br>___None	
6	Omwaaka ogu nyina bamukwatsiremu akakooko ka sirimu	_ _ _ _	
7	Enda y’okubanza	____eego<br>____ngaaha	
	**Ekicweeka kyakabiri: Okugaba omubazogwa Nevirapine**		
8	Nyina akatunga omubazi gwa nevirapine ahabwenkora ya PMTCT?	____eego<br>____ngaaha	→ **Yemereza okubuuza**
9	Akatunga nevirapine omucupa n’empitirizo ninga n’enshaho ya Pratt ninga by’ombiri?	____ ecupa (bottle and syringe)<br>____ akapapura (Pratt pouch)<br>____ byoona (both)<br>____ obujuuma (tablets)	**Ku aratorane, rekyeraho ebibuzo (If Tablets, stop interview)**
10	Akatunga ecupa ninga ebishaho bingahi buri murundi obuyabitungire?		

	ANC	L&D	PNC wiki 2 n'ahansi	PNC wiki 2 n'ah'eiguru
Ecupa				
Ebishaho				

11	Omwaana akamara wiki 6 eza nevirapine?	____eego<br>____ngaaha	
12	Omwaana ari omukabi kahango akokukwaatwa srimu?	____eego<br>____ngaaha	**Za aha kibuuzo 14**
13	Omwaana akamara wiki 12 eza nevirapine?	____ eego<br>____ngaaha	
	**BUUZA NYINA EBIBUUZO EBI**		
14	Naansi (nurse) akagambaho naiwe ahabikwatiraine n'okuha omwaana waawe omubazi gw'akakooko ka sirimu?	____ eego<br>____ngaaha	
15	Omwaana waawe okamuzarira omwirwariro?	____ eego<br>____ngaaha	**Za aha kibuuzo 17**
16	Ku araabe atarazarire mwirwariro, ninshongaki eyaretsire otazarira mwirwariro?<br><br>*Ypreka ekigarukwamu (ansa) kimwe kyonka*	____ okukwaatwa ebisha by'okuzaara mangu-mangu haza nkaremwa kuhika omwirwariro<br>____ obutagira entambura<br>____ okubura esente z'okushashura entambura n'eirwariro<br>____ okwenda kuzarira ow'omuzarisa<br>____ omushaija wangye obutabaho<br>____ nkaba nyine enshaho ya Pratt<br>____ omushaija wangye akanzibira<br>___ ebindi<br>Byoreke aha______________	
17	Omumibazi eyibakuhaire omucupa/enshaho/ninga byombiri, haine eyiwahaire omwaana waawe?	____ eego<br>____ngaaha	**Za aha kibuuzo 19**
18	Yaaba kiri kityo, omwaana okamuhera emibazi ebiro bingahi?	____ (ebiro)	
19	N'ebiro bingahi ebi otarahaire mwaana omubazi?	____ (ebiro)	
	**Reeba ekyagarukwamu aha kibuuzo nnamba p, yaaba ni "ecupa y'omka". Yemereza ebibuuzo**		
20	Ebishaho ebitakozesibwe (ebitigwirwe) osigize bingahi?	_____(ennamba y'ebishaho)<br>____tinkumanya	

21	Orakoziseho enkora ya PMTCT?	____ eego<br>____ngaaha	
22	N'okunda muringo ki ogw'okukuherezamu emibazi y'omwaana waawe?	_____ecupa n'empitirizo<br>_____ enshaho	
23	Okatunga bizibu ki omukukozesa enshaho?	___ tikiriho<br>___ okugiigura<br>___ obutetegyereza endagiriro y'okukozasa enshaho<br>___ okweebwa endagiriro<br>___ okugibiika<br>___ obuhango bw'enshaho<br>___ nkaremwa kuteeka omubazi omukanwa k'omwaana<br>___ okwontsya kukanteganisa omukuha omwaana omubazi<br>___ ebindi<br>Byoreke aha____________________	

Webare kwetaba omubibuuzo ebi!
